# Immune cell infiltration as a biomarker for the diagnosis and prognosis of stage I–III colon cancer

**DOI:** 10.1007/s00262-018-2289-7

**Published:** 2018-12-19

**Authors:** Rui Zhou, Jingwen Zhang, Dongqiang Zeng, Huiying Sun, Xiaoxiang Rong, Min Shi, Jianping Bin, Yulin Liao, Wangjun Liao

**Affiliations:** 1 Department of Oncology, Nanfang Hospital, Southern Medical University, 1838 North Guangzhou Avenue, Guangzhou, 510515 People’s Republic of China; 2 Department of Cardiology, Nanfang Hospital, Southern Medical University, Guangzhou, Guangdong People’s Republic of China

**Keywords:** Immune risk score, Colon cancer, Diagnosis, Prognosis, CIBERSORT

## Abstract

Tumour-infiltrating immune cells are a source of important prognostic information for patients with resectable colon cancer. We developed a novel immune model based on systematic assessments of the immune landscape inferred from bulk tumor transcriptomes of stage I–III colon cancer patients. The “Cell type Identification By Estimating Relative Subsets Of RNA Transcripts (CIBERSORT)” algorithm was used to estimate the fraction of 22 immune cell types from six microarray public datasets. The random forest method and least absolute shrinkage and selection operator model were then used to establish immunoscores for diagnosis and prognosis. By comparing immune cell compositions in samples of 870 colon cancer patients and 70 normal controls, we constructed a diagnostic model, designated the diagnostic immune risk score (dIRS), that showed high specificity and sensitivity in both the training [area under the curve (AUC) = 0.98, *p* < 0.001] and validation (AUC 0.96, *p* < 0.001) sets. We also established a prognostic immune risk score (pIRS) that was found to be an independent prognostic factor for relapse-free survival in every series (training: HR 2.23; validation: HR 1.65; entire: HR 2.01; *p* < 0.001 for all), which showed better prognostic value than TNM stage. In addition, integration of the pIRS with clinical characteristics in a composite nomogram showed improved accuracy of relapse risk prediction, providing a higher net benefit than TNM stage, with well-fitted calibration curves. The proposed dIRS and pIRS models represent promising novel signatures for the diagnosis and prognosis prediction of colon cancer.

**Electronic supplementary material:**

The online version of this article (10.1007/s00262-018-2289-7) contains supplementary material, which is available to authorized users.

## Introduction

Colon cancer is one of the major human malignancies. Although progress in surgical techniques and systemic treatments have improved the overall prognosis of patients with colon cancer when diagnosed at an early stage [1, 2], current pathophysiological evaluation, treatment decisions, and prognostic predictions for colon cancer mainly rely on factors with a cancer cell-centric focus, such as the TNM staging system [3], and molecular markers. However, numerous studies have recently pointed to the influence of the immune microenvironment on colon cancer development [4], suggesting that infiltration of different types of immune cells might be a promising source of novel diagnostic and prognostic biomarkers.

Among the various cell types involved in cancer development and progression, the prognostic impact of tumor-infiltrating lymphocytes has been most extensively studied to date, including colon cancer. Indeed, assessment of the extent of tumor-infiltrating lymphocytes was confirmed to be an important supplemental marker to the TNM staging system for relapse and mortality prediction [5–7]. Besides lymphocytes, tumors also commonly contain diverse non-lymphocyte immune cells [8, 9], which are considered to have a unique impact on prognosis in different cancer types and disease stages [4]. However, conventional means of measuring the tumor immune infiltrate, such as IHC or flow cytometry, are not capable of comprehensively assessing the immune effects of different cell types or do not show effective discriminating power between closely related cell populations, which is largely due to the limitation of the number of immune markers that can be simultaneously measured with current techniques. As an alternative, continuously accumulating transcriptomics data can provide an ideal resource for large-scale analysis of the immune landscape, and multiple computational methods have been developed to carry out such analyses [10]. With the goal of improving early diagnosis and prognosis prediction in colon cancer, in the current study, we employed the algorithm “Cell type Identification By Estimating Relative Subsets Of RNA Transcripts (CIBERSORT)”, which has been deemed to be the most accurate method available. CIBERSORT is an algorithm that allows for highly sensitive and specific discrimination of 22 human immune cell phenotypes using a machine-learning approach called support vector regression [11] and has already been used for immunoscore model construction in several cancer types [12–14]. Here, we used CIBERSORT to quantify the proportions of immune cells in samples of 870 colon cancer patients and 70 normal controls based on their gene expression profiling available from public databases. We also developed two novel immune-based models to provide more powerful biomarkers for the diagnosis and prognosis of colon cancer patients.

## Materials and methods

### Colon cancer datasets and normal controls

We searched the Gene Expression Omnibus (GEO; http://www.ncbi.nlm.nih.gov/geo/) for eligible datasets that fulfilled the following criteria: included samples were hybridized to the HG-U133A (GEO accession number GPL96) or Affymetrix HG-U133 Plus 2.0 (GEO accession number GPL570) platforms; more than 50 patients were included in each dataset, and information on the TNM stage was available. The raw “CEL” files of the microarray data were downloaded and normalized using a robust multiarray averaging method [15] with “affy” and “simpleaffy” packages. The mRNA expression profiles of non-tumoral colon mucosas that were included with the eligible colon cancer datasets served as non-malignant (normal) controls. They were from the corresponding tumor patients of the cohort we analyzed. These mRNA data will be called normal controls.

### CIBERSORT estimation

The gene expression data with standard annotation were uploaded to the CIBERSORT web portal (http://cibersort.stanford.edu/), and the algorithm was run using the LM22 signature and 1000 permutations [11]. Cases with a CIBERSORT output of *p* < 0.05, indicating that the inferred fractions of immune cell populations produced by CIBERSORT are accurate [16**]**, were considered to be eligible for further analysis. For each sample, the final CIBERSORT output estimates were normalized to sum up to one and thus can be interpreted directly as cell fractions for comparison across different immune cell types and datasets. The optimal cut-off values for a fraction of each immune cell type were defined as the point with the most significant (log-rank test) split, and calculated using the web-based tool “cutoff Finder” (http://molpath.charite.de/cutoff/) [17] for the entire cohort.

### Study population and clinicopathological variables

The samples were randomly separated into training and validation (7:3) sets for diagnostic and prognostic analyses based on cohorts for identifying and evaluating the models using the “caret” package. The following clinical information was collected from the databases: patients’ age, sex, TNM stage, and primary tumor site. Data on the microsatellite instability (MSI) status, chromosome instability status, genetic mutations (*KRAS, BRAF*, and *P53*), and consensus molecular subtypes (CMS) [18], specifically microsatellite instability immune (CMS1), canonical (CMS2), metabolic (CMS3), and mesenchymal (CMS4), were also retrieved where available. To maintain consistency among the dataset, the TNM stage of all patients was converted to that defined by the 6th edition [19]. The endpoint analyzed in this study was relapse-free survival (RFS), defined as the interval between the date of diagnosis and date of tumor relapse.

### Gene set enrichment analysis

Gene set enrichment analysis (GSEA) [20] was used to investigate the potential mechanisms in the “Molecular Signatures Database” of c2 (c2.cp.kegg.v6.1.symbols and c2.cp.biocarta.v6.1.symbols) and c5 (c5.bp.v6.1.symbols) using the JAVA program (http://software.broadinstitute.org/gsea/index.jsp). The number of random sample permutations was set at 1000, and the significance threshold was set at *p* < 0.05.

### Statistical analysis

All statistical analyses were conducted using R software (version 3.4.0) and SPSS software (version 25.0). Missing values were handled by multiple imputation analyses [21]. Group comparisons were performed for continuous variables using the independent *t* test for normally distributed variables and Mann–Whitney *U* test for variables showing an abnormal distribution. The correlations between the immunoscore value and mRNA expression level of corresponding genes were analyzed using Spearman’s correlation test. Random forest analysis and least absolute shrinkage and selection operator (LASSO) analysis were both applied to identify the most important immune cells that could be used to differentiate tumor and normal tissues. The overlapping markers between these two methods were finally selected to build the diagnostic prediction model using a logistic regression method [22]. Survival rates were calculated by the Kaplan–Meier method, and significance of differences between survival curves was determined using the log-rank test. Uni- and multivariate analyses were performed using Cox proportional hazard models. The LASSO–Cox method was implemented to reduce the dimensionality and to select the most significantly relapse-associated immune cells to build a prognostic model using the Cox regression method [23]. Nomogram construction and validation were performed according to Iasonos’ guide [24]. The sensitivity and specificity of the diagnostic and prognostic prediction models were analyzed by receiver operating characteristic (ROC) curve and time-dependent ROC [25] curve, respectively, and quantified by the area under the ROC curve (AUC). The discrimination of the prognostic models was measured and compared by Harrell’s concordance index (c-index). All statistical tests were two sided and *p* values less than 0.05 were considered statistically significant. This study was conducted and reported in line with the Transparent Reporting of a Multivariate Prediction Model for Individual Prediction or Diagnosis guidelines [26].

## Results

### Patient characteristics

Data of a total of 870 patients diagnosed with stage I–III colon cancer from six GEO datasets (GSE17536, GSE33113, GSE37892, GSE38832, GSE41258, and GSE39582) were retrospectively analyzed in this study. The median age at diagnosis was 68.0 years (range 22.0–96.0 years) and 420 (48.3%) of the patients were male. Detailed patient characteristics are listed in Supplemental Table 1. The patient selection scheme and workflow chart are shown in Supplemental Fig. 1.

### Composition of immune cells in tumor and normal tissue

**Fig. 1:**
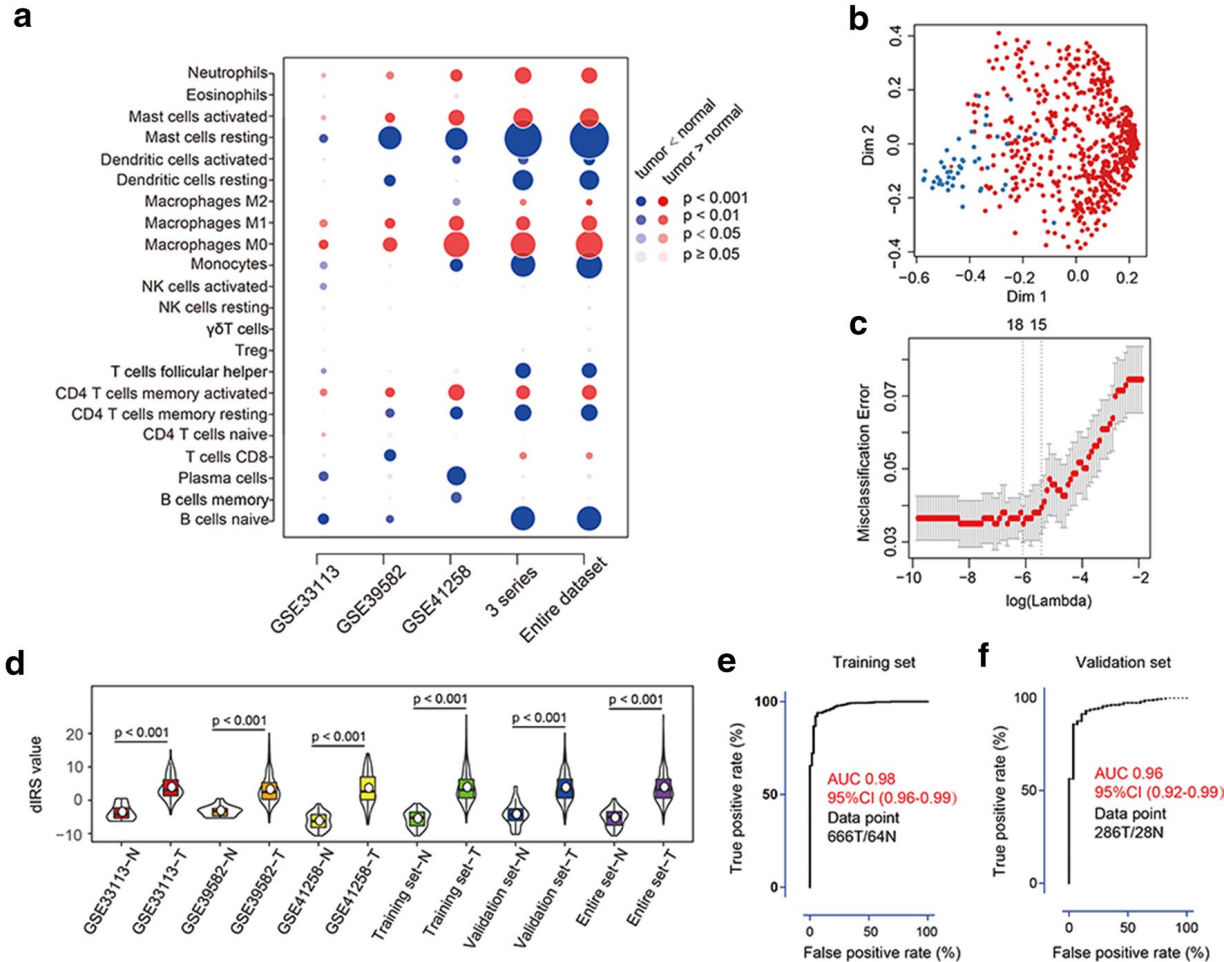
dIRS construction and validation. **a** Bubble heatmap for comparison of the immune cell fraction difference between tumor and normal colon tissues. Fractions of each immune cell type were compared by means of a two-sided Mann–Whitney *U* test for colon cancer. A red circle indicates a higher immune cell-type fraction in colon cancer as compared with normal colon tissue. A blue circle indicates a lower fraction in colon cancer as compared with normal colon tissue. The size of the circle represents the absolute value of the Z statistics. **b** Multi-dimensional scaling plot of a proximity matrix generated from random forest analysis in the training cohort. The blue dots represent normal samples and the red dots indicate tumor samples. *Dim* dimension. **c** Misclassification error for different numbers of variables revealed by the LASSO regression model. The red dots represent the value of misclassification error, the grey lines represent the standard error (SE), the two vertical dotted lines on the left and right, respectively, represent optimal values by the minimum criteria and 1-SE criteria. “Lambda” is the tuning parameter. **d** Distribution of dIRS values in different datasets. The box plots inside the violin indicate the median value and interquartile range of dIRS. The white points represent mean dIRS values. *dIRS* diagnostic immune risk score; *N* normal; *T* tumor. **e, f** ROC of the dIRS model in the training (**e**) and validation (**f**) cohorts. *AUC* area under curve; *CI* confidence interval; *N* normal; *T* tumor

We first analyzed the composition of immune cells in colon cancer tissues versus normal colon tissues. As shown in Fig. 1a, the fractions of activated CD4+ memory T cells, M0 and M1 macrophages, activated mast cells, and neutrophils were consistently higher in the colon cancer tissue than those of the normal tissue, whereas only the fraction of resting mast cells was significantly lower in all series in the colon cancer tissue. A summary of the immune cell composition within and across clinical subgroups of colon cancer tissues further showed that plasma cells, M2 macrophages, CD4+ resting memory T cells, M0 macrophages, and activated mast cells were the five most common immune cell fractions, and the sum of their mean proportions was more than 60% in all clinical subgroups (Supplemental Fig. 2).

### Immune cells for diagnostic prediction of colon cancer

We separated patients into training and validation cohorts (Supplemental Table 2), and found no significant difference in baseline characteristics between the two groups (all *p* > 0.05; Supplemental Table 2). The random-forest analysis (Fig. 1b) and LASSO analysis (Fig. 1c) revealed eight overlapping markers between the two methods. Using a logistic regression method, we established a diagnostic immune risk score (dIRS) model with these markers (Supplemental Table 3). In this model, the fractions of selected immune cells were evaluated as continuous variables. The violin plot (Fig. 1d) showed that the dIRS value was significantly upregulated in colon cancer tissues in each gene expression series in both the training and validation cohorts, and in the entire patient cohort. The dIRS model also showed high accuracy in distinguishing colon cancer patients from normal controls (Fig. 1e, f). In addition, we evaluated the ability of dIRS in differentiating between colon polyps and cancer. Similarly, a significant difference in the dIRS value was observed between these two diseases (Supplemental Fig. 3a). The dIRS model showed over 80% sensitivity and specificity for differentiating colon cancer patients from those with polyps (Supplemental Fig. 3b, c).

### Immune cells for the prognostic prediction of colon cancer

**Fig. 2:**
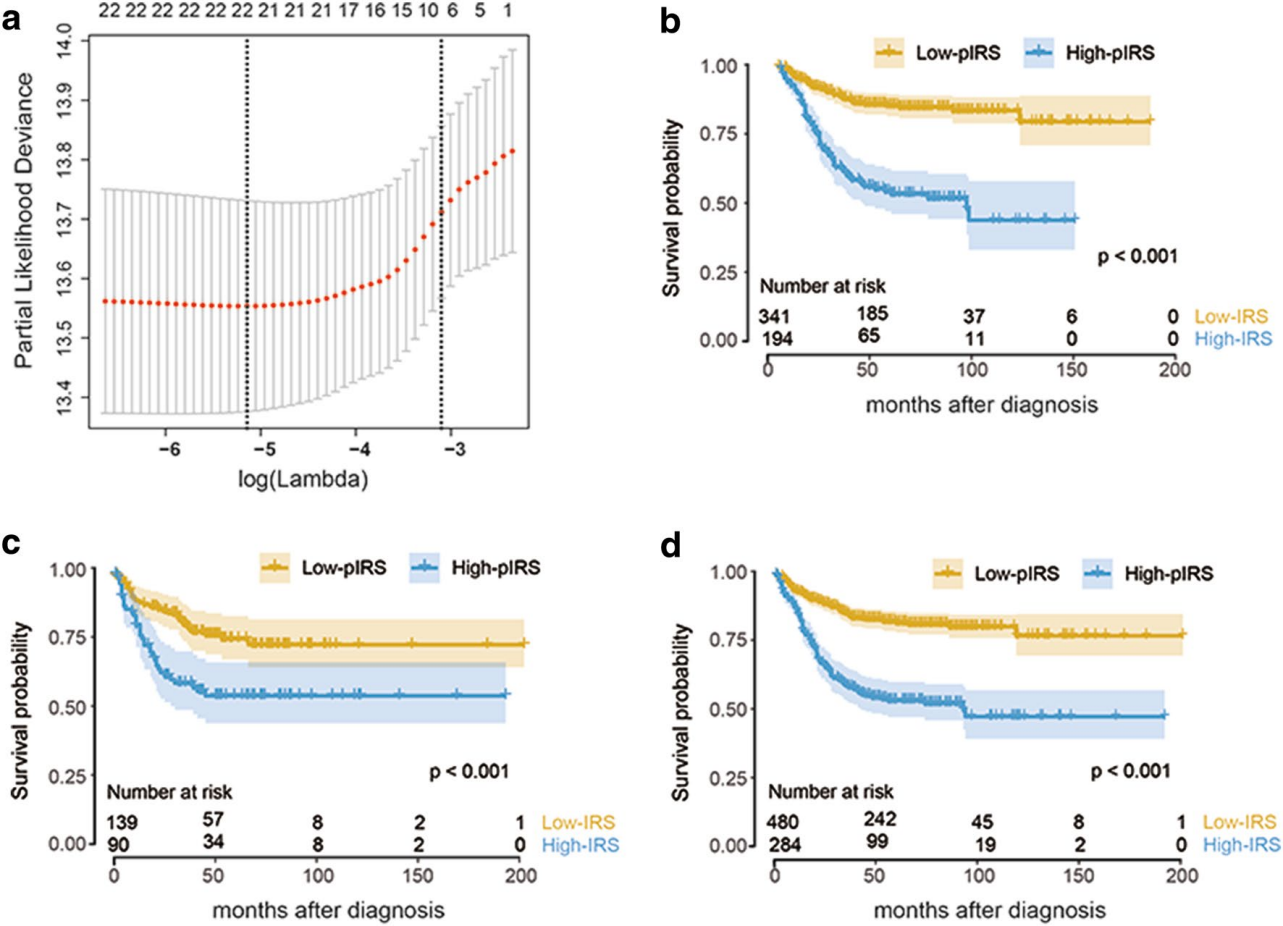
pIRS construction and validation. **a** Partial likelihood deviance of different numbers of variables revealed by the LASSO regression model. The red dots represent the partial likelihood deviance values, the grey lines represent the standard error (SE), the two vertical dotted lines on the left and right, respectively, represent optimal values by minimum criteria and 1-SE criteria. “Lambda” is the tuning parameter. **b**–**d** Kaplan–Meier curves of relapse-free survival according to pIRS groups in the training cohort (**b**), validation cohort (**c**), and entire cohort (**d**). *pIRS* prognostic immune risk score

Five of the six datasets (GSE17536, GSE33113, GSE37892, GSE38832, and GSE39582) evaluated, in which the samples were all hybridized to GPL570, were used for prognostic model construction and patients were randomly regrouped into training and validation cohorts for this purpose (Supplemental Table 2). The cut-off values for each cell type are listed in Supplemental Table 4. Through the LASSO algorithm (Fig. 2a), 22 types of immune cells were selected to build the prognostic immune risk score (pIRS) model using Cox analysis in the training cohort (Supplemental Table 5), and the predictive ability of the pIRS at 2, 3, and 5 years was represented by AUC values (Supplemental Table 6). In this model, the cell fraction was converted into binary variables, and was given a value of 1 or 2 to represent a value higher or lower than the cut-off value as described in our previous study [12]. According to the cut-off value obtained in the entire cohort (4.74), we divided the patients into high- or low-pIRS groups. The Kaplan–Meier curves suggested that the patients in the high-pIRS group had a significantly higher risk of relapse in the training set (HR 3.90, 95% CI 2.70–5.61, *p* < 0.001), validation set (HR 2.25, 95% CI 1.38–3.68, *p* < 0.001), and entire set (HR 3.22, 95% CI 2.41–4.31, *p* < 0.001) by the log-rank test (Fig. 2b–d). The pIRS was also found to be a strong independent risk factor for survival through multivariate analysis when treated as a continuous variable in all patient cohorts (Table 1).

**Table 1:** Univariate and multivariate survival analyses of pIRS and clinical variables

	UVA		MVA					
	Entire	*p* value	Training	*p* value	Validation	*p* value	Entire	*p* value
Age^a^	1.00 (0.99–1.01)	0.542	1.00 (0.99–1.02)	0.753	1.00 (0.98–1.02)	0.761	1.00 (0.99–1.01)	0.709
Gender (vs. male)	0.73 (0.55–0.98)	**0.036**	0.66 (0.45–0.97)	**0.032**	0.81 (0.48–1.36)	0.419	0.72 (0.53–0.97)	**0.031**
pIRS^a^	2.51 (2.11–2.98)	< **0.001**	2.23 (1.77–2.81)	< **0.001**	1.65 (1.12–2.45)	< **0.001**	2.01 (1.75–2.52)	< **0.001**
Tumor site (vs. proximal)	0.95 (0.71–1.29)	0.775	1.02 (0.67–1.53)	0.942	1.08 (0.61–1.91)	0.795	1.06 (0.76–1.49)	0.728
Stage (vs. stage I)								
Stage II	7.63 (1.88-31.00)	**0.004**	6.39 (0.87–46.71)	0.068	3.10 (0.42–23.00)	0.268	5.08 (1.24–20.80)	**0.024**
Stage III	13.70 (3.38–55.52)	< **0.001**	11.63 (1.59–89.84)	**0.016**	3.65 (0.48–27.69)	0.210	7.91 (1.93–32.44)	**0.004**
CMS subtype (vs. CMS4)								
CMS1	0.60 (0.37–0.98)	**0.041**	0.84 (0.47–1.52)	0.562	0.78 (0.31–2.00)	0.602	0.83 (0.49–1.41)	0.475
CMS2	0.49 (0.34–0.70)	< **0.001**	0.54 (0.33–0.87)	**0.011**	0.89 (0.44–1.80)	0.740	0.66 (0.45–0.95)	**0.027**
CMS3	0.49 (0.28–0.85)	**0.011**	0.46 (0.21–0.97)	**0.042**	1.07 (0.40–2.86)	0.885	0.64 (0.36–1.15)	0.137

Bold values indicate *p* < 0.05

*pIRS* prognostic immune risk score, *UVA* univariate analysis, *MVA* multivariate analysis; *CMS* consensus molecular subtypes

^a^Continuous variable

Since the information on MSI status, chromosome instability status, and genetic mutations could only be retrieved from the GSE39582 series, we specifically explored whether the pIRS model maintained its survival impact when the above variables were simultaneously regarded as concomitant variables (Supplemental Table 7). Similarly, the pIRS was still significantly negatively associated with RFS either through univariate analysis (*p* < 0.001) or multivariate analysis (*p* < 0.001).

We next performed stratification analyses in various subgroups for the entire cohorts, where the pIRS was treated as a continuous variable. As shown in Supplemental Table 8, the pIRS identified patients with different prognoses in all subgroups analyzed. In the c-index analysis (Table 2), the pIRS model showed better predictive ability than that of the TNM stage in the training, validation, and entire cohorts.

**Table 2:** Harrell’s concordance indexes of the pIRS, stage, and nomogram in different cohorts

Cohort	pIRS	Stage 6^th^	Nomogram
Training	0.72 (0.68–0.76)	0.60 (0.54–0.66)	0.71 (0.63–0.77)
Validation	0.67 (0.61–0.74)	0.54 (0.46–0.62)	0.64 (0.55–0.73)
Entire	0.70 (0.66–0.74)	0.59 (0.55–0.64)	0.69 (0.64–0.74)

*pIRS* prognostic immune risk score

### Nomogram construction

**Fig. 3:**
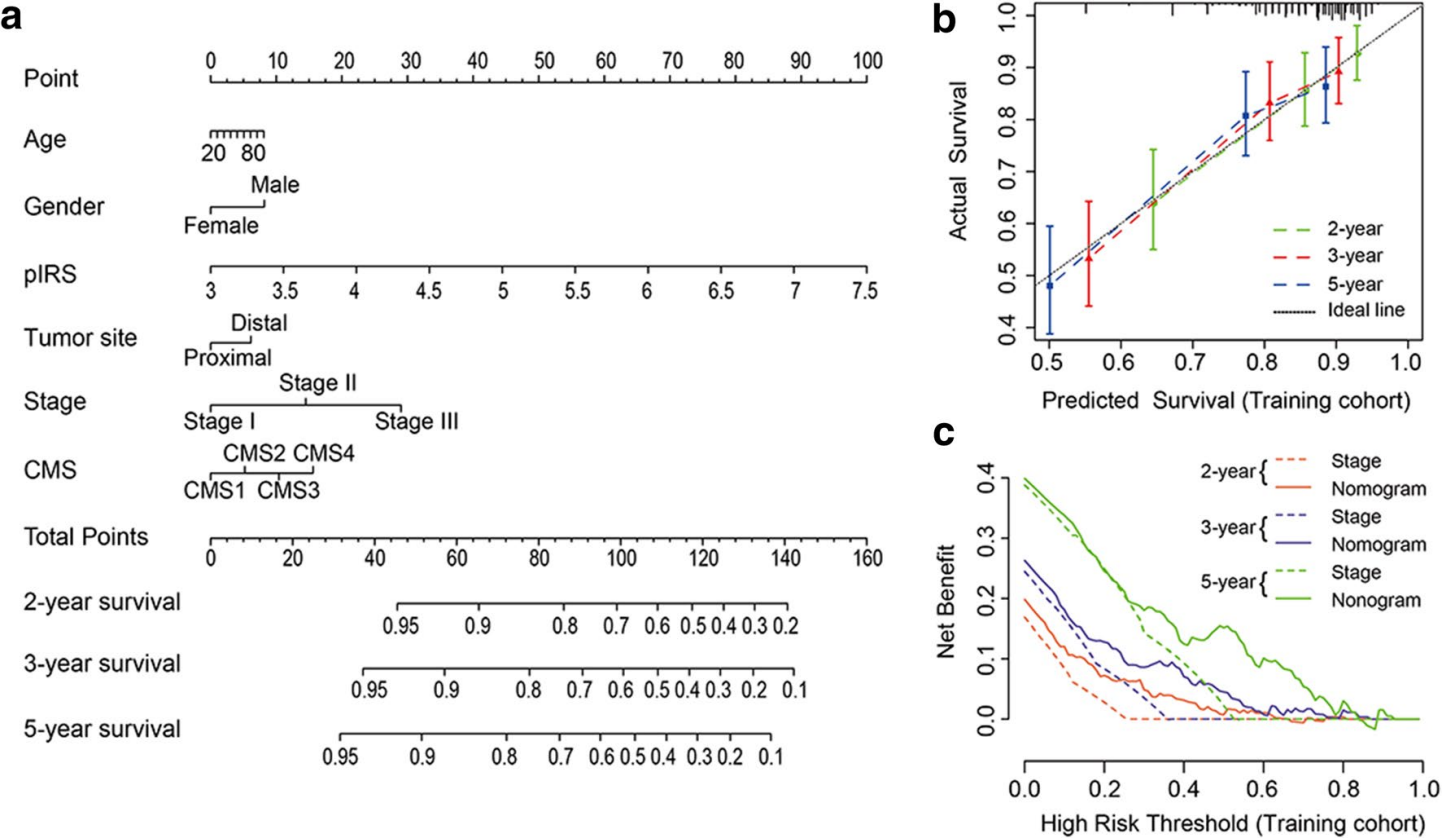
Nomogram construction and validation. **a** Nomogram for predicting 2-, 3-, and 5-year RFS for colon cancer patients in the training cohort based on pIRS and clinicopathological parameters. *pIRS* prognostic immune risk score; *CMS* consensus molecular subtypes. **b** Calibration curves of nomograms in terms of agreement between predicted and observed 2-, 3-, and 5-year outcomes in the training cohort. The dashed line of 45° represents perfect prediction, and the actual performances of our nomogram are shown by green, red, and blue lines. **c** Decision curve analyses of the nomogram and TNM stage for 2-, 3-, and 5-year risk in the training cohort

To provide a quantitative method to predict the probability of relapse, we constructed a nomogram that integrated both the pIRS and clinicopathological factors using patients from the training cohort (Fig. 3a, Supplemental Table 9). The calibration plots depicted in Fig. 3b and Supplemental Fig. 4a, b showed that the derived nomogram performed well when compared to the performance of an ideal model using the training cohort, validation cohort, and entire cohort. Similarly, using the decision curve (Fig. 3c and Supplemental Fig. 4c, d) and c-index analysis (Table 2), the nomogram also showed a higher net benefit and better predictive accuracy than the TNM staging system.

### Correlations between pIRS with clinical characteristics and molecular subtypes

**Fig. 4:**
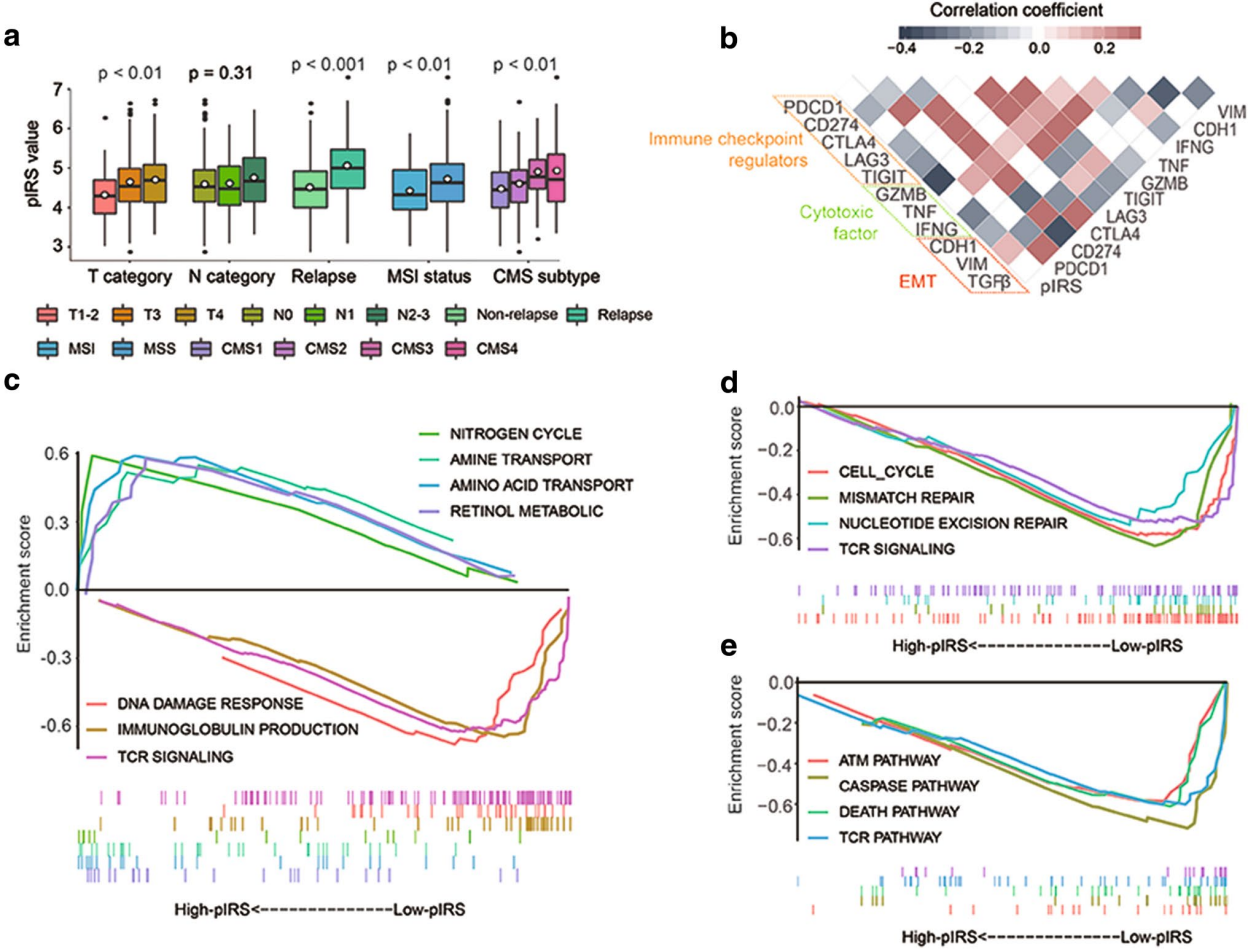
Clinical significance and biological function of pIRS. **a** pIRS values in different clinical subgroups. *pIRS* prognostic immune risk score; *CMS* consensus molecular subtypes; *MSI* microsatellite instability; *MSS* microsatellite stable. **b** Correlation matrix of pIRS values and the expression levels of certain genes. The shade of colour reflects the value of corresponding correlation coefficients. *pIRS* prognostic immune risk score; *EMT* epithelial–mesenchymal transition. **c**–**e** Gene set enrichment analysis delineates biological pathways and processes correlated with pIRS values using gene sets of “c5.bp.v6.1.symbols” (**c**), “c2.cp.kegg.v6.1.symbols” (**d**), and “c2.cp.biocarta.v6.1.symbols” (**e**) downloaded from the MSigDB database. Samples were classified into high- and low-pIRS groups. Each run was performed with 1000 permutations. Enrichment results with significant associations between high- and low-pIRS groups are shown. *pIRS* prognostic immune risk score

The correlations between pIRS with clinical characteristics and molecular subtypes were further investigated in the GSE39582 series. As shown in Fig. 4a, apart from the lymph node metastatic status, pIRS significantly varied between patients with different tumor invasion levels, relapse occurrence status, and MSI status. Additionally, in terms of molecular subtypes, patients in CMS3 and CMS4 exhibited significantly higher pIRS values, whereas CMS1 was notably linked to a low pIRS.

### Biological phenotypes associated with the pIRS model

Gene expression data were analyzed to explore the potential biological phenotypes associated with the pIRS model. First, we specially focused on the correlation between pIRS and the expression of selected immune-related genes. The heatmap depicted in Fig. 4b shows that the pIRS was significantly negatively correlated to the expression levels of *PD-L1* (*p* < 0.001), *LAG3* (*p* < 0.001), *TIGHI* (*p* < 0.001), *GZMB* (*p* < 0.001), and *IFNG* (*p* < 0.001). Interestingly, the pIRS was also correlated with markers of epithelial–mesenchymal transition (EMT). Finally, we performed GSEA to elucidate the biological functions of the pIRS model (Fig. 4c–e), which revealed that genes highly expressed in the low-pIRS group showed significant enrichment in multiple biological processes such as cell cycle regulation, DNA repair, cell apoptosis, cell death, and immune activation pathways, while the high-pIRS-related genes were associated with the metabolism-related gene set, including retinol metabolism, nitrogen cycle, and amine and amino acid transport organization.

## Discussion

Although it has long been recognized that the immune context plays an important role in tumor initiation and development [4, 27], these insights have not had a major influence on routine clinical practice. Moreover, the role of genes that are aberrantly expressed in cancer tissue on diagnosis and prognosis has attracted substantial interest; however, very few of these studies focused on the difference of the composition of immune cells between cancer and normal tissues. In the present retrospective study, we first established a diagnostic prediction model (dIRS) based on the fractions of eight types of immune cells. The significant stepwise increase in the dIRS value from a normal colon to polyp and to tumor tissue, as well as the high AUC value not only demonstrated that the dIRS model could effectively identify patients with colon cancer from individuals with colon polyps and healthy controls but also suggested that the immune system participates in colonic carcinogenesis. This finding opens the door to a new diagnostic strategy from the perspective of immune infiltration. Nevertheless, future studies are warranted to establish the consistency between immune cells in the circulation and their infiltration status in the tissues to determine whether the immune patterns detected in peripheral blood could be used as a novel tool for colon cancer screening.

Tumor relapse after initial resection is one of the most important factors influencing the total survival of colon cancer patients. Therefore, accurate assessment of patient relapse risk is essential for improving personalized cancer care. To date, studies on the prognostic role of the density of CD3+ and CD8+ lymphocytes in the central- and peri-tumoral areas represented by intensity of IHC staining have gained increased attention [4, 27, 28], and this method has been validated through both a single-centre cohort study [5] and international multi-centre validation in localized colon cancer [6]. However, the assessment of only CD3+ and CD8+ lymphocytes cannot comprehensively reflect the local immune status. Technically, IHC suffers from limitations in available phenotypic markers and can, therefore, be challenging to practically implement and standardize. Instead, the use of transcriptomics data to describe the tumor microenvironment computationally is a promising approach that overcomes the technical limitations of IHC, and can further characterize diverse immune populations with multiple functional phenotypes in a large patient cohort much more readily than possible with IHC. Therefore, by applying the newly developed algorithm “CIBERSORT”, our pIRS model differs from previously reported immune models that consist of features of the lymphocytes and myeloid cells simultaneously. Subsequent c-index analyses and subgroup analyses further confirmed the prognostic ability and excellent reproducibility of pIRS for colon cancer. However, according to the guidelines established by Altman et al. [29], only signatures validated in independent cohorts of patients with full clinical annotation available could be applied clinically. Therefore, we will first validate the prognostic value of pIRS model at our centre and compare the prognostic value of the pIRS and IHC-based immunoscore model in a same cohort in future studies. Since the current high-throughput gene expression measurement technology has been well developed, we believe that our pIRS classifier has strong potential to be translated into clinical practice.

We also uncovered a significant difference in the pIRS value among CMS subtypes, with a higher value in the CMS3 and CMS4 subtypes than in the CMS1 and CMS2 subtypes. Profound biological differences were demonstrated among distinct CMS groups. Among them, CMS3 showed enrichment for multiple metabolism signatures. This is consistent with the GSEA result showing that a high pIRS value was correlated with biological processes related to metabolism. Moreover, the enrichment of EMT-related genes in CMS4 was also supported by the correlation between the pIRS value and EMT marker genes. By contrast, the pIRS value was the lowest in the CMS1 subtype, which is characterized by increased expression of genes involved in the immune response, along with an emerging feature of MSI. Notably, MSI status has been proposed as a promising predictor for the treatment efficacy of immunotherapy such as anti-PD-1 treatment [30]. Since our study also revealed significant variation of the pIRS value between patients with different MSI status, as well as obvious enrichment of multiple immune checkpoint markers, inflammatory factors, and immune activation pathways in the low-pIRS group, it is reasonable to speculate that immunotherapy might also be a preferable choice for patients in this group. Further studies are warranted to explore whether the pIRS model can predict the response of patients with colon cancer to immunotherapy.

There are inevitably several limitations of our study that should be acknowledged. First, the amount of data released in publicly available datasets is limited, so that the clinicopathological parameters analyzed in this study are not comprehensive, which might lead to potential error or bias. Second, we have not considered the heterogeneity of the immune microenvironment related to the location of immune infiltration. Third, all data series downloaded for establishment of the dIRS and pIRS models were from Western countries and all transcriptome profiling was produced by the GPL96 or GPL570 platform; thus, caution should be exerted when applying the conclusion of this study to patients from Asian countries and samples tested using platforms other than GPL96 or GPL570. Finally, microarray data are generally considered to not be clinically practical. Thus, reducing the dIRS and pIRS to assays that are appropriate for clinical application will be another important task in our future work.

In conclusion, our study demonstrates the utility of consideration of immune cells in the diagnosis, treatment evaluation, and prognosis of stage I–III colon cancer. The proposed dIRS and pIRS models might provide much needed comprehensive clinical information for improving the personalized management of colon cancer patients.

## Electronic supplementary material

Below is the link to the electronic supplementary material.

Supplementary material 1 (PDF 7703 KB)

## Abbreviations

AUC: Area under the curve
CIBERSORT: Cell Type Identification By Estimating Relative Subsets Of RNA Transcripts
c-index: Concordance index
CMS: Consensus molecular subtypes
dIRS: Diagnostic immune risk score
EMT: Epithelial–mesenchymal transition
GEO: Gene expression omnibus
GSEA: Gene set enrichment analysis
LASSO: Least absolute shrinkage and selection operator
MSI: Microsatellite instability
MSS: Microsatellite stability
pIRS: Prognostic immune risk score
RFS: Relapse-free survival
ROC: Receiver operating characteristic
