# Supplementary material for: Immune cell infiltration as a biomarker for the diagnosis and prognosis of stage I–III colon cancer
Source: Cancer Immunol Immunother. 2018 Dec 19;68(3):433–42. doi: 10.1007/s00262-018-2289-7 (PMC6426802; doi:10.1007/s00262-018-2289-7)
Supplement: Supplementary file 1 — Supplementary material 1 (PDF 7703 KB) [file 262_2018_2289_MOESM1_ESM.pdf]

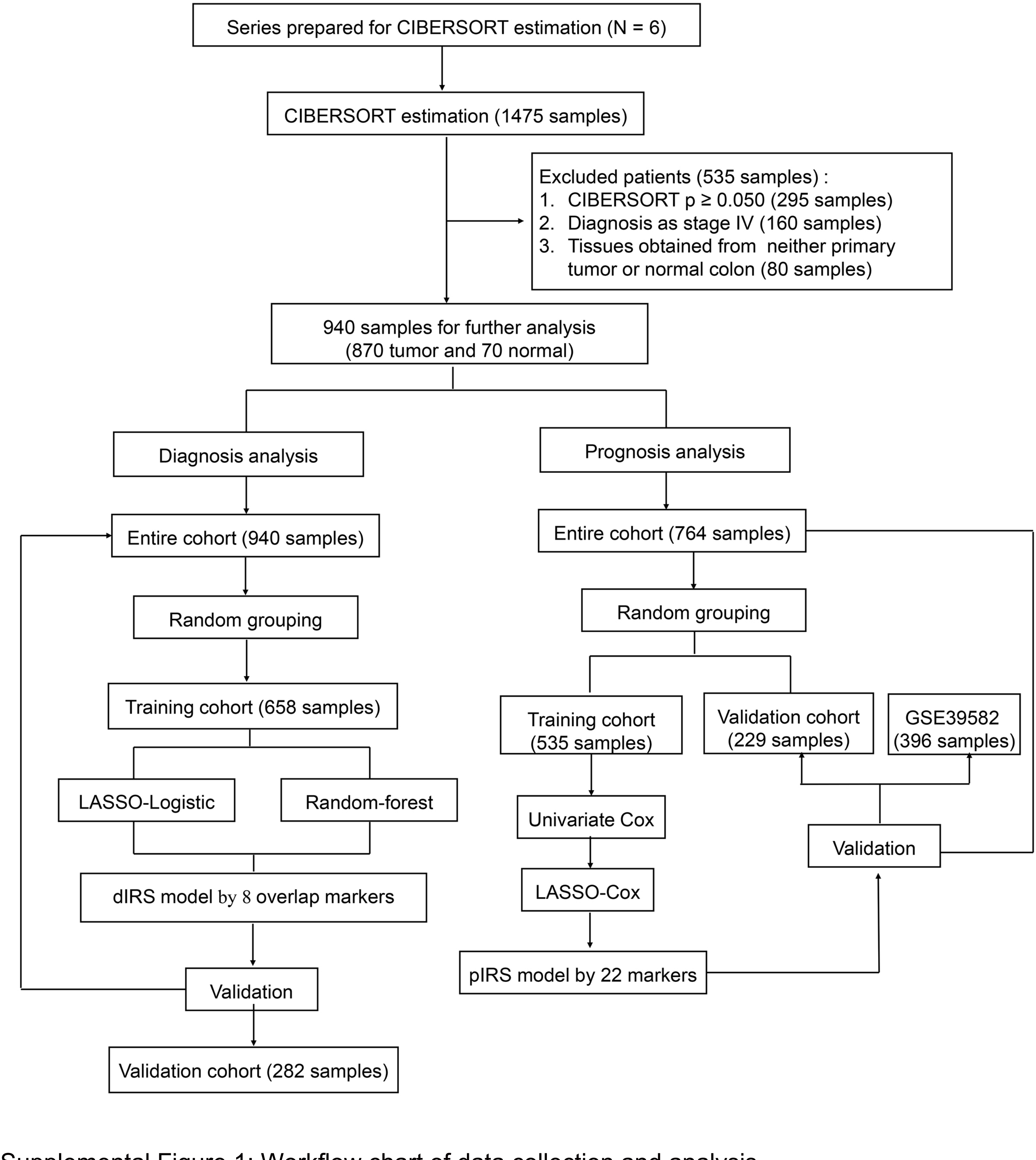

Supplemental Figure 1: Workflow chart of data collection and analysis

CIBERSORT,Cell type Identification By Estimating Relative Subsets Of RNA Transcripts; LASSO, least absolute shrinkage and selection operator; dIRS, diagnostic immune risk score; pIRS, prognostic immune risk score.

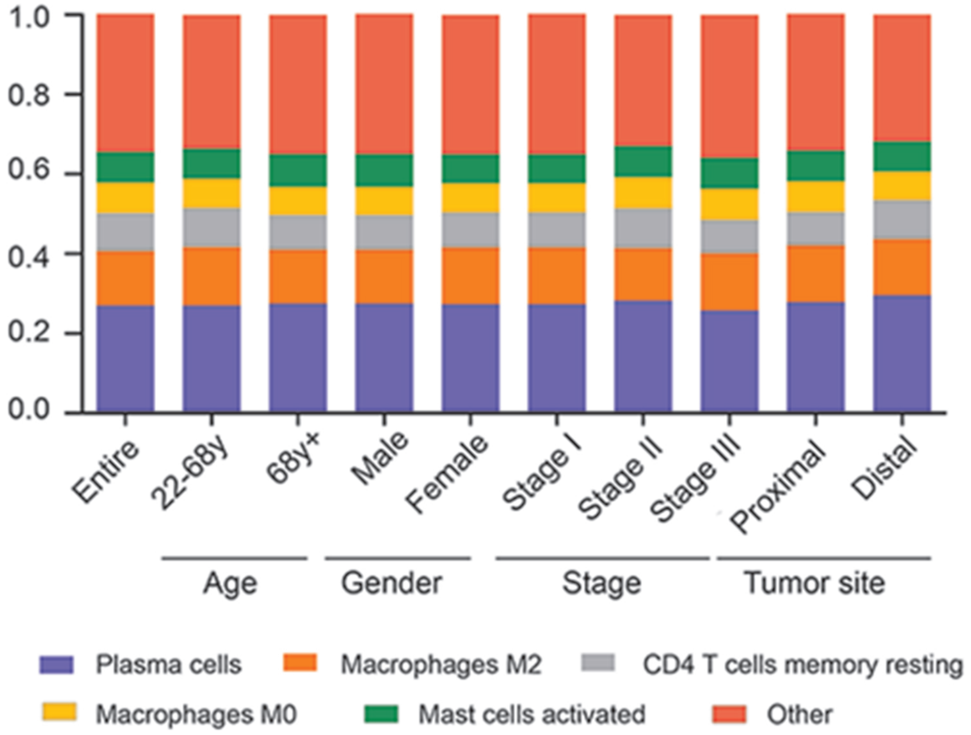

Supplemental Figure 2: Summary of inferred immune cell subsets.

Bar charts summarizing immune cell subset proportions within and across clinical subgroups of colon cancer tissues in the entire cohort.

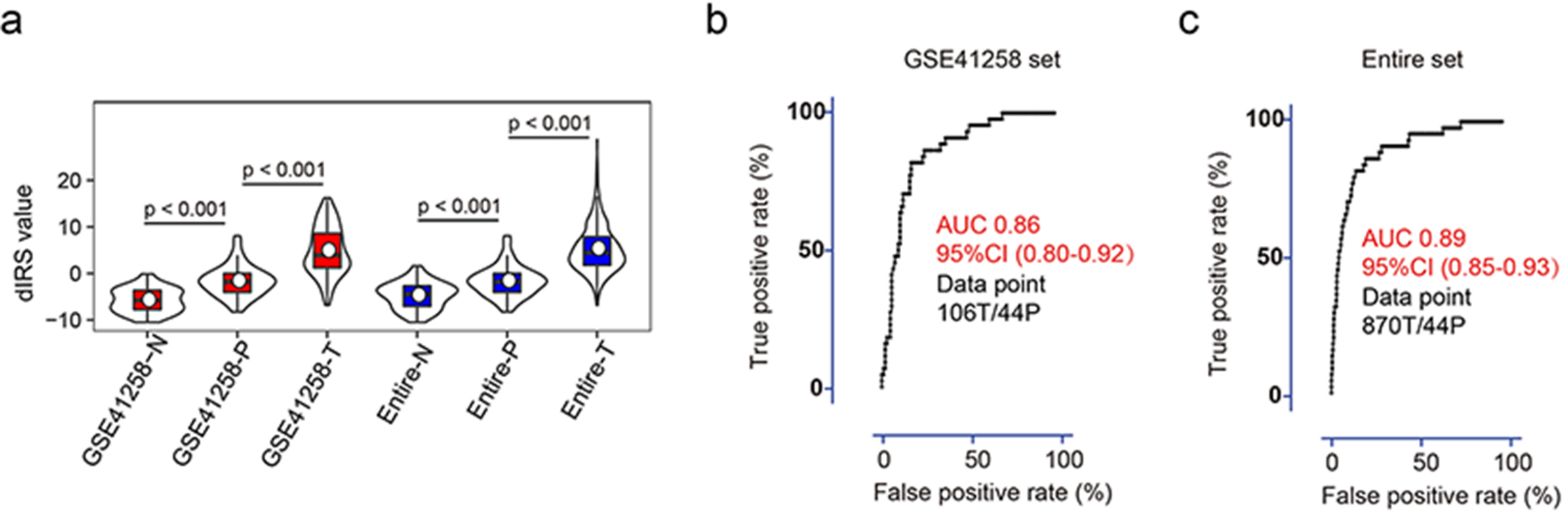

Supplemental Figure 3: dIRS values in colonic polyp and tumour samples.

(a) Distribution of dIRS values in colonic polyp and tumour tissues. The box plots inside the violin indicate the median values and interquartile ranges of dIRS. The white points represent mean dIRS values; (b,c) Receiving operator characteristic curve and AUC values of the dIRS model for differentiating polyp and tumour samples in the training (b) and validation sets (c).*dIRS, diagnostic immune risk score; N, normal; P, polyp; T, tumour; AUC, area under curve; CI, confidence interval.*

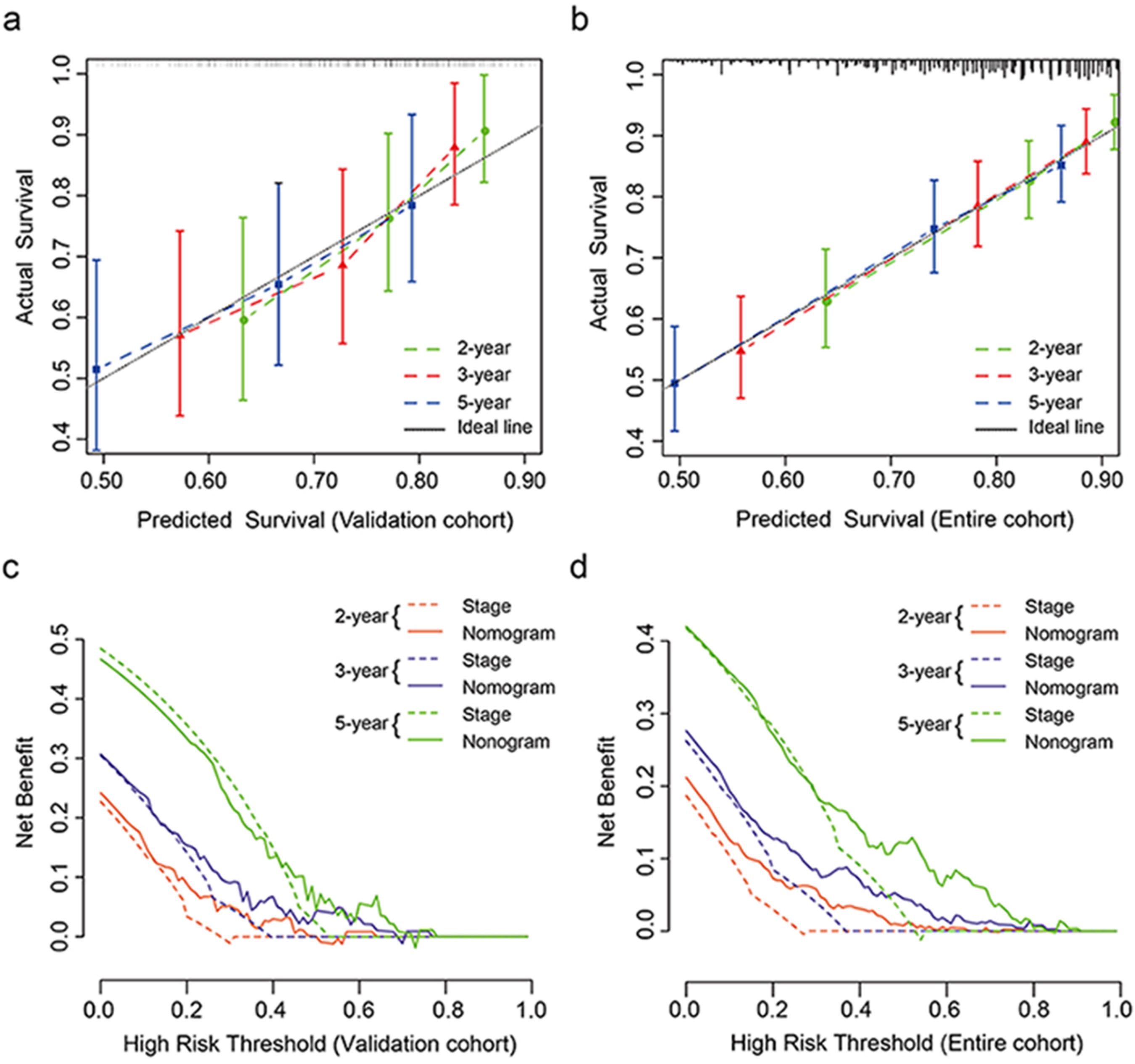

Supplemental Figure 4: Calibration curves and decision curves of the nomogram in the validation and entire cohorts.

(a,b) Plots depicting the calibration of the nomogram in terms of agreement between predicted and observed 2-year, 3-year, and 5-year outcomes in the validation(a) and entire(b) cohorts. The nomogram performance is shown by the plot, relative to the 45-degree line, which represents perfect prediction; (c,d) Decision curve analyses of the nomogram and TNM stage for 2-year, 3-year, and 5-year risk in the validation (c) and entire (d) cohorts.

Supplemental Table 1. Patients' basic characteristics

Item		Number	Percent (%)
**Age**	22-68	406	46.7
	> 68	391	44.9
	Unknown	73	8.4
**Gender**	Male	420	48.3
	Female	379	43.6
	Unknown	71	8.7
**Stage**	I	90	10.3
	II	449	51.6
	III	331	38.0
**Tumor site**	Proximal	251	28.9
	Distal	333	38.3
	Unknown	286	32.9
**CMS subtype**	CMS1	129	14.8
	CMS2	248	28.5
	CMS3	81	9.3
	CMS4	173	19.9
	Unknown	239	27.5
**Total**		870	

Abbreviation: *CMS, consensus molecular subtypes*

Supplemental Table 2. Comparison of demographics and clinical variables of the training and validation cohorts

Item	Diagnosis analysis			Prognosis analysis		
	Training cohort (n = 609)	Validation cohort (n = 261)	P value	Training cohort (n = 535)	Validation cohort (n = 229)	P value
**Age** (years; mean ± SD)	66.90 ± 12.42	66.89 ± 13.08	0.99	66.97 ± 12.77	67.70 ± 12.93	0.50
**Male** (n, %)	296 (53.1)	123 (51.2)	0.64	257 (48.0)	109 (47.6)	0.99
**Stage**			0.13			0.41
I	58 (9.5)	32 (12.5)		49 (9.2)	18 (7.9)	
II	328 (53.6)	119 (46.5)		278 (52.0)	131 (57.2)	
III	226 (36.9)	105 (41.0)		208 (38.9)	80 (34.9)	
**Tumor site (**proximal, **%)**	178 (44.4)	72 (39.8)	0.32	180 (27.0)	84 (29.5)	0.42
**CMS subtype**			0.68			0.92
CMS1	88 (19.7)	41 (22.2)		93 (17.4)	36 (15.7)	
CMS2	178 (39.9)	70 (37.8)		166 (31.0)	82 (35.8)	
CMS3	54 (12.1)	27 (14.6)		56 (10.5)	25 (10.9)	
CMS4	126 (28.3)	47 (25.4)		126 (23.6)	47 (20.5)	

Abbreviation: *CMS, consensus molecular subtypes; SD, standard deviation.*

Supplemental Table 3. Coefficients of immune cell markers in colon cancer diagnosis

Immune cell marker	Coefficients	SE	P value
Naïve B cells	-28.83	6.00	< 0.001
T cells follicular helper	-29.97	8.54	< 0.001
Monocytes	49.32	19.01	0.009
Macrophage M0	41.50	11.06	< 0.001
Macrophage M1	41.28	11.68	< 0.001
Dendritic cells resting	-25.89	9.72	0.008
Mast cells resting	-32.68	8.23	0.000
Mast cells activated	8.12	7.13	0.225

Abbreviation: *SE, standard errors*

Supplemental Table 4. Cut-off values for immune cell fractions

Cell type	Cut-off value
B cells naive	0.0803
B cells memory	0.0835
Plasma cells	0.0587
T cells CD8$^+$	0.0615
CD4$^+$ T cells naive	0.0351
CD4$^+$ T cells memory resting	0.2426
CD4$^+$ T cells memory activated	0.0246
T cells follicular helper	0.0832
T cells regulatory	0.0779
γδT cells	0.0758
NK cells resting	0.0911
NK cells activated	0.0342
Monocytes	0.0012
Macrophages M0	0.0228
Macrophages M1	0.0314
Macrophages M2	0.2277
Dendritic cells resting	0.0436
Dendritic cells activated	0.0402
Mast cells resting	0.0767
Mast cells activated	0.1820
Eosinophils	0.0580
Neutrophils	0.0408

Supplemental Table 5. Coefficients of immune cell markers in colon cancer prognosis

Immune cell marker	Coefficients	SE	P value	HR	Lower CI	Upper CI
B cells naive	-0.65	0.52	0.212	0.52	0.19	1.45
B cells memory	0.31	0.24	0.209	1.36	0.84	2.19
Plasma cells	-0.69	0.32	0.029	0.50	0.27	0.93
T cells $CD8^+$	0.31	0.21	0.137	1.36	0.91	2.04
$CD4^+$ T cells naive	-0.26	0.39	0.506	0.77	0.36	1.66
$CD4^+$ T cells memory resting	1.01	0.43	0.02	2.76	1.18	6.41
$CD4^+$ T cells memory activated	-0.15	0.22	0.509	0.86	0.56	1.34
T cells follicular helper	-0.44	0.55	0.422	0.66	0.22	1.88
T cells regulatory	0.65	0.53	0.218	1.92	0.68	5.44
γδT cells	1.13	0.62	0.07	3.08	0.91	10.43
NK cells resting	-0.83	0.61	0.178	0.44	0.13	1.45
NK cells activated	0.11	0.40	0.776	1.12	0.52	2.43
Monocytes	0.48	0.21	0.019	1.62	1.08	2.43
Macrophages M0	-0.14	0.21	0.49	0.86	0.57	1.31
Macrophages M1	-0.48	0.19	0.013	0.62	0.42	0.90
Macrophages M2	0.70	0.27	0.01	2.00	1.18	3.40
Dendritic cells resting	0.14	0.28	0.618	1.15	0.66	2.01
Dendritic cells activated	0.60	0.39	0.131	1.80	0.84	3.87
Mast cells resting	1.23	0.45	0.007	3.41	1.40	8.28
Mast cells activated	0.21	0.41	0.605	1.24	0.55	2.76
Eosinophils	1.27	0.54	0.017	3.57	1.25	10.22
Neutrophils	0.66	0.25	0.008	1.94	1.19	3.16

Abbreviation: *SE, standard errors, HR, hazard ratio; CI, confidence interval.*

Supplemental Table 6. Time-dependent receiver operator characteristic (ROC) analysis of pIRS

Cohort	Time point	AUC (95%CI)
Training cohort	2-year	75.21 (69.76-80.65)
	3-year	75.35 (70.08-80.62)
	5-year	74.11 (68.31-79.91)
Validation cohort	2-year	72.05 (63.81-80.28)
	3-year	69.14 (60.86-77.42)
	5-year	65.67 (56.39-74.95)
Entire cohort	2-year	74.03 (69.45-78.90)
	3-year	73.48 (69.02-77.93)
	5-year	71.61 (66.69-76.53)

Abbreviation: *ROC, receiver operating characteristic; pIRS, prognostic immune risk score; CI, confidence interval; AUC, area under curve.*

Supplemental Table 7. Univariate and multivariate survival analyses of pIRS and clinical variables in the GSE39582 series

Item	UVA		MVA	
	HR (95% CI)	P value	HR (95% CI)	P value
Age[a]	1.01 (0.99-1.03)	0.223	1.01 (0.99-1.03)	0.112
Gender (vs. Male)	0.62 (0.42-0.91)	0.016	0.56 (0.37-0.84)	0.005
pIRS[a]	2.50 (1.96-3.19)	< 0.001	2.43 (1.85-3.19)	< 0.001
Tumor site (vs. proximal)	1.04 (0.71-1.51)	0.853	1.21 (0.76-1.92)	0.418
Stage (vs. stage I)				
Stage II	8.10 (1.12-58.62)	0.038	6.09 (0.83-44.78)	0.076
Stage III	12.18 (1.69-87.90)	0.013	7.61 (1.02-56.68)	0.048
KRAS (vs. WT)	1.32 (0.91-1.92)	0.150	1.68 (1.07-2.63)	0.025
BRAF (vs. WT)	0.99 (0.52-1.90)	0.975	1.75 (0.79-3.89)	0.167
P53 (vs. WT)	1.42 (0.84-2.41)	0.177	1.52 (0.86-2.67)	0.141
MSI status (vs. MSS)	0.55 (0.30-1.00)	0.050	0.77 (0.26-2.27)	0.634
CIN (vs. positive)	1.38 (0.71-2.68)	0.336	0.84 (0.41-1.72)	0.633
CMS subtype (vs. CMS4)				
CMS1	0.42 (0.23-0.78)	0.006	0.75 (0.28-2.00)	0.558
CMS2	0.61 (0.38-0.96)	0.035	0.76 (0.46-1.25)	0.277
CMS3	0.65 (0.34-1.23)	0.187	0.66 (0.33-1.31)	0.233

[a] Continuous variable

Abbreviation: *pIRS, prognostic immune risk score; UVA, univariate analysis; MVA, multivariate analysis; HR, hazard ratio; CI, confidence interval; WT, wild type; MSI, microsatellite instability; MSS, microsatellite stability; CIN, chromosome instability; CMS, consensus molecular subtypes.*

Supplemental Table 8. Subgroup analyses of pIRS in the entire cohort

Subgroup	HR (95%CI)	P value
GSE series		
GSE17536	2.27 (1.50-3.44)	< 0.001
GSE33113	3.12 (1.85-5.28)	< 0.001
GSE37892	2.05 (1.27-3.33)	0.003
GSE38832	6.41 (2.08-19.82)	0.001
GSE39582	2.50 (1.96-3.19)	< 0.001
Age		
22-68 years	2.53 (2.01-3.19)	< 0.001
> 68 years	2.47 (1.89-3.22)	< 0.001
Gender		
Male	2.43 (1.94-3.05)	< 0.001
Female	2.55 (1.94-3.35)	< 0.001
Tumor stage		
Stage I	10.78 (1.15-101.20)	0.038
Stage II	2.59 (1.99-3.38)	< 0.001
Stage III	2.11 (1.68-2.66)	< 0.001
Tumor site		
Proximal	2.30 (1.80-2.93)	< 0.001
Distal	2.77 (2.10-3.63)	< 0.001
CMS subgroup		
CMS1	2.29 (1.48-3.53)	< 0.001
CMS2	2.13 (1.42-3.18)	< 0.001
CMS3	3.97 (1.89-8.35)	< 0.001
CMS4	2.34 (1.83-3.00)	< 0.001

Abbreviation: *pIRS, prognostic immune risk score; HR, hazard ratio; CI, confidence interval; CMS, consensus molecular subtypes.*

Supplemental Table 9. Cox regression coefficients and nomogram scores

Cohort		Coefficients	Score
Age[a]		0.001	0.10 * Age - 2.03
Gender	Male		8.17
	Female	-0.42	0
pIRS[a]		0.79	22.22*pIRS-66.67
Tumor site	Proximal		0
	Distal	0.12	6.14
Stage	I		0
	II	1.28	14.51
	III	1.85	29.02
CMS subtype	CMS1		0
	CMS2	-0.14	5.20
	CMS3	-0.28	10.41
	CMS4	0.38	15.61

[a]Continuous variables

Abbreviation: *pIRS, prognostic immune risk score; CMS, consensus molecular subtype.*
